# The 1926 novel, “One, no one, one hundred thousand”, metaphorizes the potential danger when the immune system is exposed to a repetitive antigen stimulation

**DOI:** 10.3389/fimmu.2023.1254853

**Published:** 2023-09-13

**Authors:** Francesca Ferrazzo, Sara Leto, Natalia Malara

**Affiliations:** Department of Health Sciences, University Magna Graecia, Catanzaro, Italy

**Keywords:** idiotypic/anti-idiotypic network, vaccine, SARS-CoV-2, cancer vaccine, precision medicine

## Abstract

In the worldwide scenario of infection prevention and control, the vaccine strategies are destined to increase rapidly. The availability of numerous vaccination options allows you to plan individually on how to boost your immune system. The immune system is a highly plastic cognitive dynamic network and performs its function by recognition of the uniqueness of the organism defined as self. The identification and attack of non-self antigens contribute to improving the strategies of self/non-self discrimination. However, repetitive antigen stimulation of the immune system may lead to several outcomes reassumed in three principal risks: (i) loss of the unique self codification (one), (ii) loss of own identifying (no one), and (iii) the increase of idiotype/anti-idiotype entities (one hundred thousand). Controlled production of idiotype/anti-idiotype antibodies protects against autoimmune diseases and immunodeficiency. The title of the famous novel by Nobel Prize for Literature winner Luigi Pirandello, “One, no one, one hundred thousand”, recaps the three risks and the protagonist’s journey exploring the complexities of personal identity, and warns to preserve the uniqueness of the organism. Taking inspiration from this metaphor, the authors propose to monitor antibody idiotype response for personalizing vaccine plans with the aim of preserving the uniqueness of the immune system and assuring safe protection.

## Introduction

The immune system is a highly plastic cognitive dynamic network that performs its function through the recognition of the uniqueness or molecular identity of the organism defined as self. The antigen constitutes the input signal that interrogates the immune system engaging cellular and humoral components related to its specific processing. The immune system in the first instance catalogs the outside molecular as belonging (self) or not belonging (non-self) to the organism. Once the antigen’s non-self nature is ensured, the immune system generates an adequate response to its elimination. Therefore, it expands its network with a new immune clone competent to recognize, bind, and specifically eliminate the non-self entity (1). The final dimension of the response network as well as its expanding architecture depends on timing, concentration, and the type of antigenic stimulation. A high concentration stimulation of antigen in a short space of time generates an equally intense immune response. The intensity of the immune response is determined by the extension of the network related to the number of cellular elements and molecular entities enrolled. Moreover, its architecture is determined by the type of antigen that conditions the kind of immune elements engaged (2). The heterologous cell connectivity between the immune cell and antigen and the homologous connectivity between the immune cell and the immune cell are factors conditioning the dimension, the archetype, and the control system of the response immune (3). The expansion and duration of the immune response are limited in time and in space. The immune system itself performs the function of supervision and control through negative feedback. This function consists in generating in parallel, during the interaction with the antigen, a network called idiotype/anti-idiotype. According to Niels Jerne’s Network Theory, the immune system is a network of interacting idiotypes involved in the regulation of immune responses (1). The term idiotype for determinants recognized by antibodies was adopted, recognizing that antibodies against antibodies exist and playing a number game on the multitude of B cells producing antibodies. Each antibody constitutes a small set of idiotopes that form its idiotype (4). In 1972, several reports appeared on the potential of anti-Id antibodies to suppress a specific immune response and that immune modulation is part of an antigen-induced immune response.

An idiotypic cascade was perceived: Ab1 > Ab2 > Ab3. Ab3 would resemble Ab1. Based on this concept, Ab2s structurally resemble the antigen, thus the term internal image of antigen for this mimicry (5).

**Figure 1:**
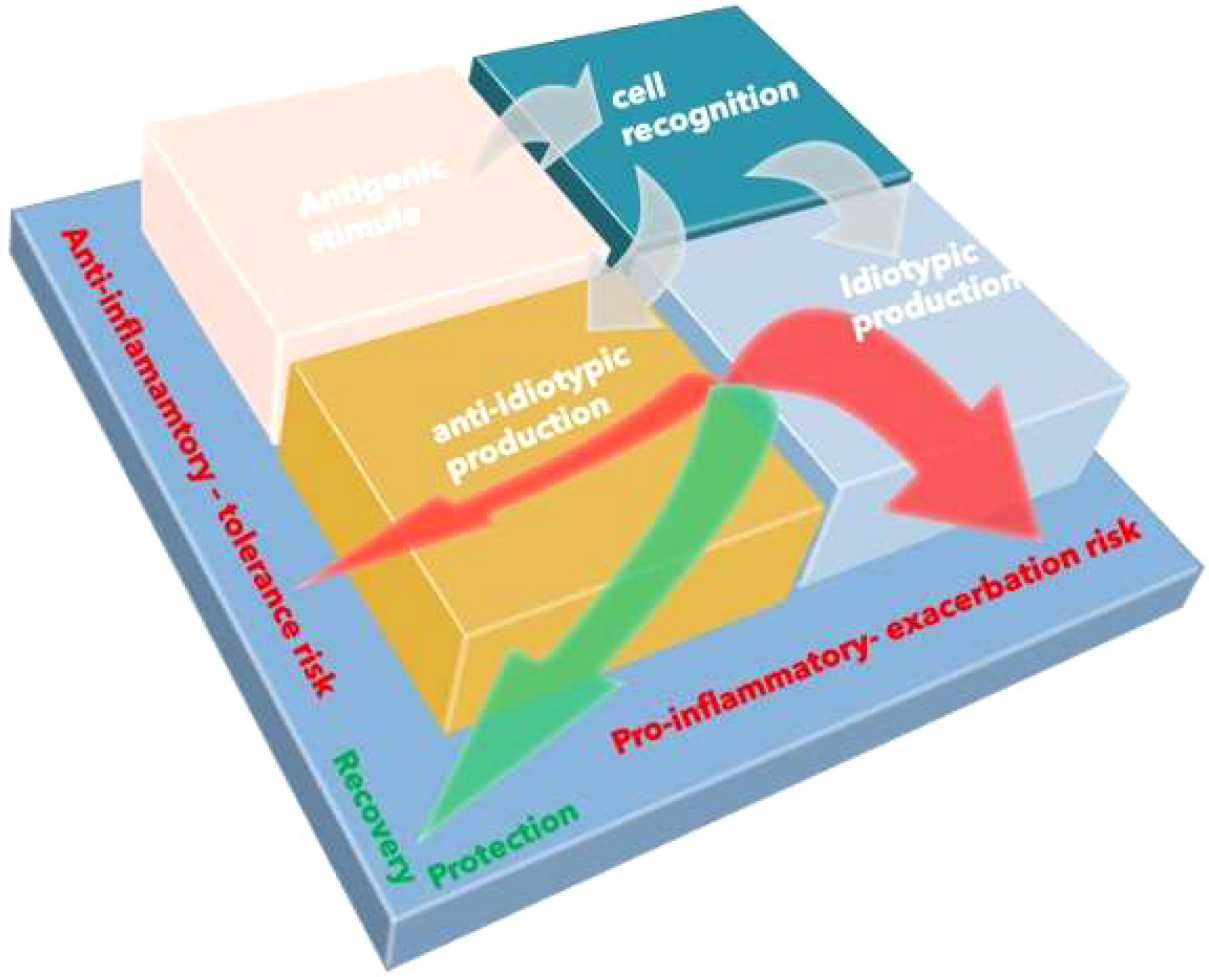
The immune circuit. The immune response induced by antigen includes molecular, antibody production, and cell engagement. The acute phase of antigen stimulation is characterized by exacerbated inflammation. Simultaneously, the immune system initiates anti-inflammatory mechanisms to reduce inflammation and promote immune memory. The exaggerated systemic release of cytokines may induce pro-inflammatory conditions; on the other hand, the activation of modulatory pathways may induce excessive tolerance promoting immunoparalysis.

Anti-idiotypic antibodies (Ab2) are a particular set of antibodies because they can react with idiotopes, i.e., the unique antigenic determinants present on the surface of an antibody. A single idiotope can span a part of the complementarity-determining regions and a part of the framework region, as well as both light- and heavy-chain residues (6). These molecules generate a network of molecular connectivity that performs the dual-containing function exercised both at the cellular level (through the T cell receptor) and the molecular level (antibodies) (7) with a dual purpose. The first purpose, in the short term, consists in limiting the intensity of the immune response (extemporaneous negative feedback), while the second with long-term effects is aimed at preserving cognitive memory (8) (delayed positive feedback) (**Figure 1**).

## Non-self antigen and repetitive antigenic stimulation

The main role of each vaccine is to administer a specifically non-self antigen that the immune system does not yet know and make it able to recognize it for immediate elimination while preserving the identification codes of the self. Recently, in the context of the anti-COVID-19 measures, as reported by Murphy and Longo (8), the idiotype–anti-idiotype network could be the cause of the serious side effects after vaccine administration, such as thrombotic events and myocarditis associated with Ab2 antibody production (8). Ab2 antibodies could also mediate the neurologic effects of SARS-CoV-2 infection (9). After SARS-CoV-2 infection or vaccines, Ab2 mimicking the S1 protein of the SARS-CoV-2 virus can interact, in turn, with the ACE receptors on the cell surface.

The presence of Ab2 in subjects infected with SARS-CoV-2 has been proven (10) and the Ab2 titer was analyzed because in interacting with the ACE2 receptors, it caused a false-positive result in tests that used the ACE2 receptor for the diagnosis of an ongoing infection (11). Moreover, the Ab2 availability favors its interaction with the ACE receptor inducing not unpredictable dysfunction if properly supervised (9).

Ab2, Ab3, and Ab4 are molecules that have the purpose of amplifying the response to antagonize the non-self and memorize the encounter with the pathogen. Part of the memorization process is seroconversion. During the pandemic, the antibody titer of the IgM-to-IgG seroconversion was considered a negative prognostic indicator for the evolution of the disease (12). These data seem to support the thesis that the antibody response in SARS-CoV-2 infection played a decisive role both in the acute phase and in the long-COVID phase. Starting from these considerations, the indication to vaccinate even people who had had the previous infection allows us to consider today that this indication should have been more weighted.

A preliminary analysis would have been useful to identify those subjects to be vaccinated because despite having had the infection, they did not have an adequate antibody response. For those subjects who instead had an adequate antibody response after infection, further vaccination resulted in repetitive antigen stimulation with potential side effects (13).

Another type of repetitive antigenic stimulation is that which occurs by simultaneously vaccinating for more than one pathogen. The soldiers invited to fronts in endemic countries for pathologies are vaccinated in a short period for multiple antigens. An effective immune response to the vaccine leads to the generation of neutralizing antibodies that can prevent pathogens’ entry (14). Many inflammatory insults can alter the functionality and the responsiveness of the innate immune system in the long term (10). Long-term reprogramming depends on the rewiring of cell metabolism and epigenetic processes and they stay at the basis of induction of both innate immune memory (also termed trained immunity) and innate immune tolerance (10). Long-term shaping of the immune responses requires that the reprogramming induced by the first contact with the antigen is maintained long enough to enhance an active secondary response after a subsequent encounter with a pathogen. It has been widely described that memory lymphocytes can survive or self-renew for long periods (9). It is possible to hypothesize that every time we administer an antigen to the immune system with vaccination, it undergoes a functional restart that consists in reprogramming with immediate and long-term effects (9). Antigenic stimulation focuses on the control and programming function of the immune system in some subjects and can determine a reduction or an exacerbation of the immunological surveillance (15).

The different antibody responses could depend on an individual level of expression density of the MHC class II system haplotypes on the lymphocyte membrane or on a low binding affinity of the haplotypes with the non-self antigen (16). Those who have a low level of expression or present a low binding affinity between these molecules and the non-self antigen could develop a limited antibody response compared to the exuberant response of other subjects with a higher degree of expression or with high binding affinity. These are some of the possible combinations that determine an individual’s antibody response when encountering the non-self antigen regardless of whether this encounter is due to an infection or a vaccination (9).

A persistence of repetitive antigen stimulation of the innate immune system seems to be involved in the development of auto aggression. As reviewed by Rifkin and colleagues (17), there is an increasing number of studies on the participation of innate immunity in the immune response to autoimmune diseases. Conversely, an iImmunoparalysis involving the surveillance functions carried out by the immune system can compromise the anti-tumor protection in some subjects compared to others (18).

## Tumor antigen and multivalent antigen stimulation

The immune system is a vital determinant of cancer and shapes its trajectory. Notably, the immune reaction to cancer harbors the dual potential for suppressing or promoting cancer development and progression. This polarity of the immune response is determined, in part, by the character of the interplay between innate and adaptive immunity. On the one hand, the innate immune compartment is a necessary proponent of cancer immunity by supporting an immunostimulatory state that enables T-cell immunosurveillance (17). However, in the context of cancer, the immune infiltrate often consists of commonly polarized innate immune cells with immunosuppressive properties to orchestrate a tolerogenic niche capable of interfering with the cytotoxic potential of tumor antigen-specific T cells (19). In the interaction with the tumor, innate immunity plays a positive and negative regulatory role in adaptive immunosurveillance (20). This unpredictable behavior mimics that of tumor cells that shunt leukocytes into an immunosuppressive state and, as such, subvert the phenotypic plasticity of the immune compartment favoring disease progression (21).

In vaccination against cancer, the immune system must be educated to recognize the cancer cell, which originates from a cell of its own body, preserves the antigenic characteristics that codify the self, and therefore not recognized as foreign by the immune system.

Different is the situation of cancers with viral pathogenesis [human papillomavirus (HPV) is linked to cervical, anal, throat, vaginal, vulvar, and penile cancers, while hepatitis B virus (HBV) is linked to chronic infections leading to increased risk of liver cancer] and for whom vaccination is recommended as a preventive measure (22).

In the case of tumors that do not have viral pathogenesis, the difficulty is trying to convince the immune system to attack the tumor cells in the body. In this case, there are mainly two difficulties. The first is that the tumor cell preserves and displays self-recognized antigens. The second is that the vaccine for treating cancer rather than preventing the disease is intended to induce the immune system to attack an already existing disease. Some vaccines are made from tumor cells, parts of cells, or pure antigens (predominantly proteins exposed on the membranes of tumor cells). Sometimes, a patient’s immune cells are isolated and exposed *ex vivo* to specific antigens to create the vaccine (23).

Lately, there is a great increase in the vaccination option and different types of antigenic stimulation, comprising the use of multivalent antigens as the hybrid antigenic stimulation, are being tested to improve the immune response against cancer. The building of immune response based on its stimulation by using a “hybrid” antigen, consisting of the combination of non-self and self components as suggested by Badrinath et al. (24), could generate important side effects in clinical use. The antigen used consists of a non-self part, the bacterial ferritin, and a self part, the a3 domain. The a3 domain, present in MHC class I chain-related proteins A and B, presents an amino acid sequence homology to the a3 domains of immunoglobulins (25). The results published by Badrinath et al. attest to an effective serum antibody conversion resulting from antigenic stimulation in animal models but do not describe the type of idiotypic/anti-idiotype redundancy induced by this type of unusual combination of self and non-self proteins. The difficulty of generating specific and efficient vaccination against cancer depends on the non-recognition of the cancer cell as non-self by the immune system (26). While modifying the molecular structure, the neoplastic transformation does not determine a significant change in the antigenic profile that can trigger the cancer cell’s recognition and elimination (27). This concept justifies why the idiotype-based therapies, developed to date, have been disappointing, forcing a new look to generate an effective approach for immunotherapy. In this perspective, the idiotype network is now being re-evaluated regarding the development of effective anti-idiotype vaccines. In particular, polyclonal anti-idiotype reagents lend themselves to the proposal that polyclonal anti-idiotype vaccines will be more effective than monoclonal-based anti-idiotype vaccines. This new strategy may soon be adapted to standardize biotechnological production of therapeutic antibodies.

## Conclusions

The generation of the immune response, its rapid expansion, and its counter-regulation are the basis of the preservation of the uniqueness and individuality of the immune system. During the continuous interplay between inside and outside, the lack of recognition of the stranger or non-self constitutes the pathogenetic basis of some immunodeficiency, and the recognition of the self as non-self is the basis of inflammatory/cancer and/or autoimmune diseases (9). The dynamic and plastic and, at the same time, the rigorous recognition of the self from the non-self constitutes the base of individual health.

Furthermore, the dogma of molecular discernment is exploited in preventive medicine with the vaccination approach.

**Figure 2:**
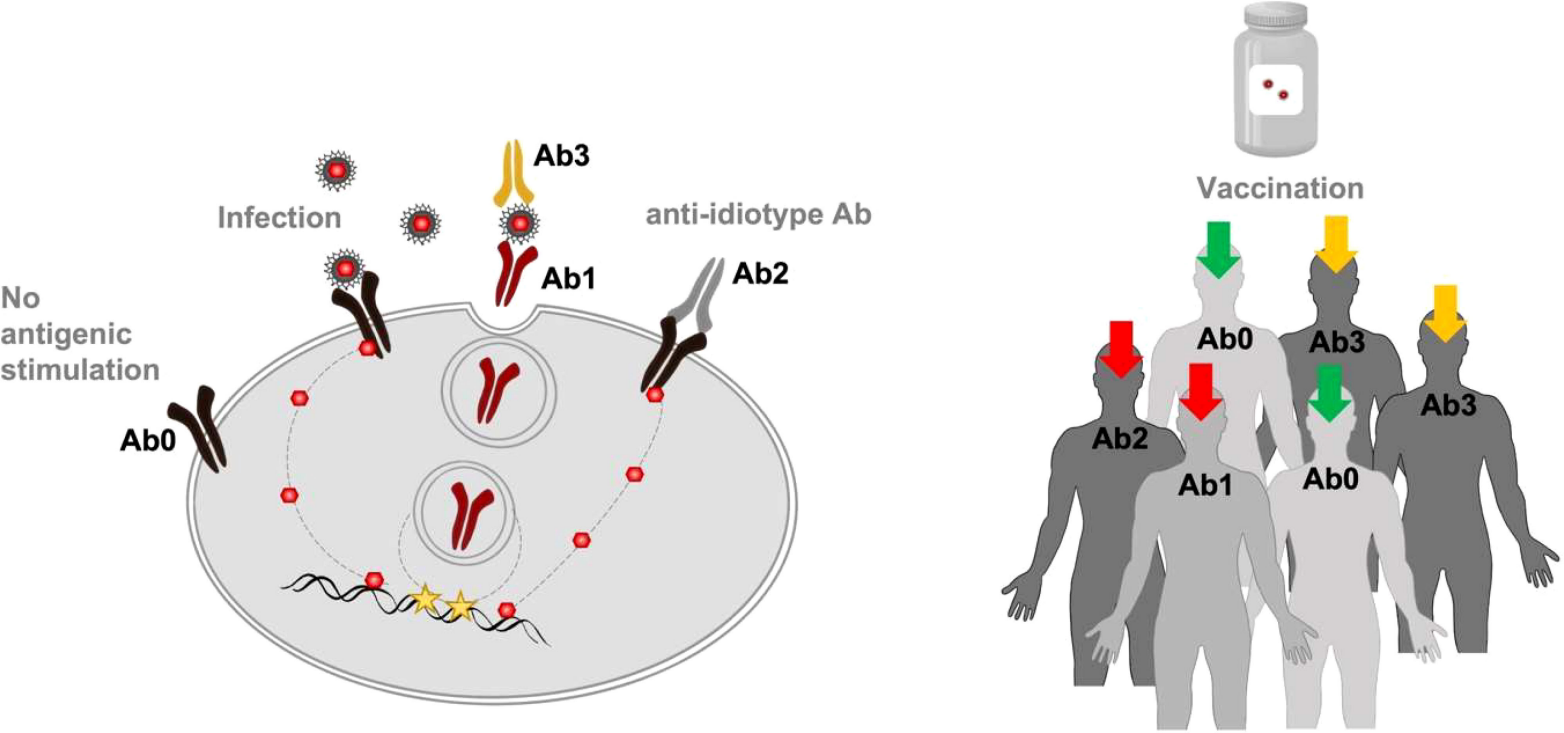
Monitoring idiotypic/anti-idiotypic network. To reduce the potential risk of immune dysfunction, a specific analysis aimed to estimate Ab2 concentration previously produced and directed against the antigen/s of interest could be performed. In particular, recognizable levels of specific Ab1 and Ab2 (red arrows) could discourage passive immunization through the vaccine. The presence of Ab3 (orange arrows) could indicate that a revaccination or a booster dose is recommended. The Ab0 condition (green arrows) could be considered as an indicator of vaccine program effectiveness/impact.

It would therefore be prudent to monitor Ab2 antibody responses to the virus and vaccines over time. Further research is needed to define the task that idiotype-based immunoregulation of humoral and cell-mediated responses performs both in the field of antipathogenic efficacy and in the sphere of undesirable effects generated during the performance of this task. The goal is to obtain a specific increase in the immune defenses for one or more pathologies without disturbing the protective immune homeostasis. For this purpose, it is advisable, before carrying out a booster vaccination, which uses the same antigenic stimulation of the first vaccination but with a lower antigenic concentration, to measure the level of Ab2 already present. To assess personalized vaccinal protection planning, it is necessary to perform a specific analysis of the Ab idiotype and anti-idiotype to prevent both humoral and cell-mediated excess responses after a vaccine. The subjects who have never met the antigen (Ab0) will benefit 100% from the administration of the vaccine; on the contrary, for the infected subjects with idiotypic Ab1 production, the vaccine is not recommended. For those subjects who have previously encountered the antigen, the indication of whether to administer the vaccine or its booster depends on the entity of their anti-idiotype Ab production (**Figure 2**).

The famous phrase in the psychological novel by Pirandello*—”*Of what I can be for me, not only can you know nothing, but nothing even myself.*”—is an important warning to keep in mind the importance of preserving the precious identifying code on uniqueness of the self (one) of the immune system from interferences or manipulations that can transform it into non-self (none) or disintegrate it into multiple idiotypic/anti-idiotypic entities (one hundred thousand), making it susceptible to dangerous decoding.In particular, the title “*One, no one, one hundred thousand”* implicitly explains the function done by the immune system, constantly engaged in protecting the cells of our organism, and the risks during the performance of his function.

*Uno nessuno centomila by Luigi Pirandello 1926 Sicily

In the psychological novel, *Vitangelo Moscarda, the protagonist, discovers from his wife that he has a crooked nose, a detail of himself that he had never noticed. This small coincidence triggers a vortex of reasoning that leads him, through various experiments, to the awareness of not being for others as he is for himself.*

## Data availability statement

The raw data supporting the conclusions of this article will be made available by the authors, without undue reservation.

## Author contributions

FF: Data curation, Writing – original draft. SL: Formal Analysis, Writing – original draft. NM: Conceptualization, Writing – original draft, Writing – review & editing, Supervision, Validation, Visualization.

## In memoriam

This paper is dedicated to the memory of Major General Marco Tuzzolino.
